# Numerical study on the impact of wall structure on the thermal performance of double-channel porous solar wall

**DOI:** 10.1038/s41598-022-19482-0

**Published:** 2022-09-07

**Authors:** Xuejun Qi, Shuang Lin, Shuyan Tao, Kumar Patchigolla

**Affiliations:** 1 School of Architecture and Civil Engineering, Xihua University, Chengdu, China; 2 School of Water, Energy & Environment, Cranfield University, Cranfield, UK

**Keywords:** Energy storage, Renewable energy

## Abstract

With the improvement of people’s living standards, they have higher requirements for indoor thermal comfort in the cold season. Solar wall utilizing solar energy for heating can reduce carbon emissions and achieve carbon neutrality. In the aspect of solar wall research, the influence of wall structure on the thermal performance of double-channel porous solar wall is limitedly investigated. In fact, the optimization design of wall structure is important for the thermal performance of solar wall and its applications. Therefore, a simplified three dimensional room model is built to study the influence of the wall structure on the thermal performance of porous solar wall by numerical simulation. With this model, different channel spacing and thickness of porous walls were used to determine the optimal design for a double-channel porous solar wall in terms of enhancing the heat storage. Moreover, the influence of the surface emissivity on the characteristics of heating and temperature field of double-channel porous solar wall are studied based on the optimal structure. The CFD simulation results indicate that the optimal structure parameters should include spacing of 0.08 m for channel 1, the porous wall thickness should be 0.08 m, and the air channel 2 spacing should be 0.06 m. The temperature of air channel 1 and air channel 2, the indoor temperature, and the heat storage of porous wall decrease with the increase of the surface emissivity of the porous wall. In order to improve the heat storage performance of double-channel porous solar wall, the outer surface of the porous wall should use a lower emissivity material. The outer surface emissivity of porous wall has a significant impact on the heat storage of the porous wall and little effect on the thermal storage wall. The temperature of porous wall is always higher than that of outdoor environment temperature.

## Introduction

With the rapid growth of the social economy and people’s requirements for indoor thermal comfort in winter, energy consumption has increased over the past years. Every year, many fossil fuels are used for heating, ventilation, and air conditioning systems. Hence, reducing energy consumption in buildings is an effective way to decrease carbon dioxide emission, which is very helpful in solving the problem of carbon emission. Trombe wall can balance the relationship between human energy demand and environmental protection due to the simple configuration, high efficiency, and low running price^1–3^.

Many researchers are working in the field of Trombe wall. A traditional Trombe wall consists of glass, an air channel, a thermal storage wall, and vents^4–6^. Abbassi et al*.* developed a numerical model to study the energetic performance of the Trombe wall in Tunisia. The results showed that the high thermal inertia wall and thermally insulated walls can significantly reduce the heating needs^7^. Compared with buildings without a Trombe wall, the buildings with such wall can save almost 20% of energy during heating season^8^. Briga-Sá et al. analyzed the Trombe wall thermal performance in different conditions of ventilation openings^9^. Liu et al. deduced that the heat storage of the Trombe wall is fully released at 7:30 in the morning. Meanwhile, the temperature decreases to its minimum value. The heat storage reached its maximum value at 16:00^10^. He et al. believed that the position of the venetian blind, the width of the air duct and the area of the inlet and outlet vents can affect the thermal performance of the Trombe wall^11,12^. Bellos et al. invented a new kind of Trombe wall with an extra window in the massive wall. The results demonstrated that this Trombe wall can enhance the indoor temperature by 0.5 K, especially during the hours between noon and afternoon^13^. Narjes and Leila reported an improved Trombe wall with a thin black copper panel. The results demonstrated that this Trombe wall increases the conductive and convective flux^14^. Gunjo et al. studied a flat plate solar water heating system under the conditions of steady state. The results showed that the thermal efficiency of the collector increases with the increase of the water flow rate, solar insolation, ambient temperature, and conductivity of the absorber plate material^15^. Ghazy et al. studied the influence of the carbon nanotube-water-nanofluid-filled Trombe wall on the heat transfer inside a typical room. They deduced that the effective conductivity of the Trombe wall is increased after the addition of nanoparticles^16^.

With the continuous development of computer software technology, numerical simulation is widely used in the field of Trombe wall^17–20^. Hong et al. built a 3-dimensional CFD model studied the flow and thermal transport in a novel Trombe wall with a venetian blind. They found that the position of the width of the air duct and the area of inlet and outlet vents affect the thermal performance of the Trombe wall^21^. Du et al. studied the impact of air flow velocity on the thermal performance of the Trombe Wall. They found that the wall structure and wall size have a close relationship with air flow velocity^22^. Bajc et al. used CFD to analyze the temperature field in the Trombe wall for several days of a typical meteorological year. The results showed that the indoor temperature is around 14.7 °C in winter^23^. Zamora et al. reported that the wall-to-wall spacing can affect the thermal and dynamic behavior of buoyancy flow^24^. Abdeen et al. investigated the optimal design for a Trombe wall in terms of enhancing thermal comfort. The results showed that the optimal structure of the Trombe wall can enhance thermal comfort by 38.19% during a typical winter week^25^.

However, in terms of Trombe wall with porous heat-storage wall, the previous study mainly focused on the particle size, porosity, thermal conductivity of porous layer, and porous absorber position in solar composite wall^26,27^, while it was concerned little about the influence of the wall structure on the thermal performance of the porous solar wall. Therefore, different simulation models are developed with different channel spacing and thicknesses of porous walls. In this study, the optimization of the wall structure is performed by Computational Fluid Dynamics. Compared with the previous research, the innovations of this paper are as follows:

1. This study deals with an optimized design of a double-channel porous solar wall. The aim is to study how the wall structure affects the thermal performance of the double-channel porous solar wall. Through the comparison of heat storage and indoor temperature, the optimal heat storage wall structure is found.
2. With the optimal structure, the influence of the surface emissivity on the air channel, indoor temperature, and heat storage is analyzed.
3. The variation of temperature field of porous wall and heat storage wall with time is also analyzed in this study.

## Numerical simulation details

### Physical model

**Figure 1:**
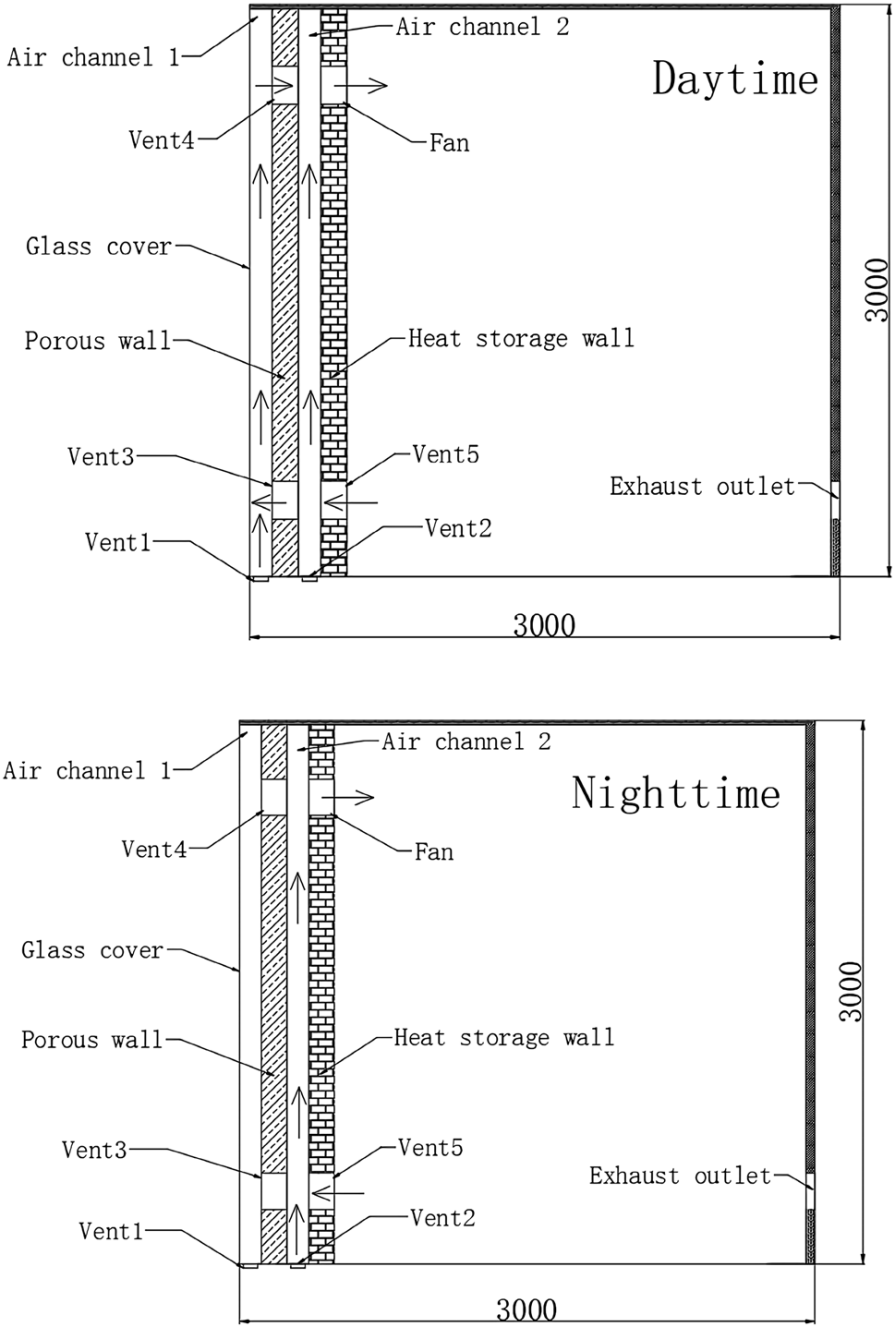
Schematic of double-channel porous solar wall.

Double-channel porous solar wall utilizes cost-free solar radiation from the sun for heating. A simplified room model is shown in Fig. 1. The size of the room is 3000 mm × 3000 mm × 3000 mm. The double-channel porous solar wall is located on the south side of the room. A transparent glass cover is installed on top of the porous wall. The size of the vent is 200 mm × 200 mm. In this system, the external surface of the porous wall absorbs solar radiation and sends thermal energy to the room for heating in the form of heat conduction, convection, and radiation. Only the fan needs to consume certain electric energy in the operation process, which significantly reduces the building energy consumption during the heating season.

During the daytime, vent 2 is closed while vents 1, 3, 4 and 5 are opened. Under the action of a fan, the mixture of fresh air from air channel 1 and indoor return air from air channel 2 is sent to the room. At night, vents 1, 3 and 4 are closed while vents 2 and 5 are opened. The mixture of indoor return air from vent 5 and fresh air from vent 2 is sent to the room through air channel 2. Simultaneously, air channel 1 becomes an air insulation layer to prevent heat loss to the environment. Consequently, a double-channel porous solar wall continues to provide heat to the room.

### Mathematical model

(1) Mass conservation equation:

1 $$\frac{\partial \rho }{{\partial t}} + \frac{{\partial (\rho u_{i} )}}{{\partial x_{i} }} = 0$$

(2) Momentum conservation equation:

2 $$\frac{\partial }{\partial t}(\rho u_{i} ) + \frac{\partial }{{\partial x_{j} }}(\rho u_{i} u_{j} ) = - \frac{\partial P}{{\partial x_{i} }} + \frac{\partial }{{\partial x_{j} }}(\mu (\frac{{\partial u_{i} }}{{\partial x_{i} }} + \frac{{\partial u_{j} }}{{\partial x_{j} }} - \frac{2}{3}\delta_{ij} \frac{{\partial u_{l} }}{{\partial x_{l} }})) + \frac{\partial }{{\partial x_{j} }}( - \rho \overline{{u_{i}^{^{\prime}} u_{j}^{^{\prime}} }} )$$

where $x_{j}$ is the Cartesian coordinates [m], $u_{j}$ is the air velocity in different directions based on Cartesian coordinates [m/s], P is static pressure [Pa], μ is molecular viscosity [Pa·s], δ_ij_ represents Kronecker delta, and $- \rho \overline{{u_{i}^{^{\prime}} u_{j}^{^{\prime}} }}$ is Reynolds stress.

3 $$- \rho \overline{{u_{i}^{^{\prime}} u_{j}^{^{\prime}} }} = \mu_{t} (\frac{{\partial u_{i} }}{{\partial x_{j} }} + \frac{{\partial u_{j} }}{{\partial x_{i} }}) - \frac{2}{3}(\rho K + \mu_{t} \frac{{\partial u_{k} }}{{\partial x_{k} }})\delta_{ij}$$

(3) Energy conservation equation^28^:

4 $$\frac{{\partial \rho \overline{{h_{s} }} }}{\partial t} + \frac{{\partial \rho \overline{{u_{i} h_{s} }} }}{{\partial x_{i} }} - \frac{{\partial \overline{P} }}{\partial t} - u_{j} \frac{{\partial \overline{P} }}{\partial t} - \overline{{u_{j} }} \frac{{\partial \overline{P} }}{{\partial x_{i} }} - \frac{\partial }{{\partial x_{i} }}(\lambda \frac{{\partial \overline{T} }}{{\partial x_{i} }}) = - \frac{\partial }{{\partial x_{j} }}(\rho (\overline{{u_{i} h_{s} }} - \overline{{u_{i} }} \overline{{h_{s} }} )$$

(4) Boussinesq assumption equation:

5 $$(\rho - \rho_{0} )g \approx - \rho_{0} \beta (T - T_{0} )g$$

(5) RNG k-ε turbulence model equation^22^:

6 $$\frac{\partial }{\partial t}(pk) + \frac{\partial }{{\partial x_{i} }}(pku_{i} ) = \frac{\partial }{{\partial x_{j} }}\left[ {\alpha_{k} \mu_{eff} \frac{\partial k}{{\partial x_{j} }}} \right] + G_{k} + G_{b} - \rho \varepsilon - Y_{M} + S_{k}$$

7 $$\frac{\partial }{\partial t}(\rho \varepsilon ) + \frac{\partial }{{\partial x_{j} }}(\rho \varepsilon u_{i} ) = \frac{\partial }{{\partial x_{j} }}\left[ {\alpha_{\varepsilon } \mu_{eff} \frac{\partial \varepsilon }{{\partial x_{j} }}} \right] + C_{1\varepsilon } \frac{\varepsilon }{k}(G_{k} + C_{3\varepsilon } G_{b} ) - C_{2\varepsilon } \rho \frac{{\varepsilon^{2} }}{k} - R_{\varepsilon } + S_{\varepsilon }$$

where *G*_*k*_ represents the average velocity gradient generated by the turbulent kinetic energy, G_b_ denotes the generation term of turbulent kinetic energy caused by buoyancy force, Y_M_ is the influence of the fluctuating expansion of compressible turbulence on the total dissipation rate, and α_k_ and α_ε_ are the effective Prandtl values of k and ε, respectively.

To simplify the calculation, the following assumptions are made in the simulation:

1. Solar radiation is the only heat source for room heating;
2. All the physical parameters of the materials in the model are constant except for air;
3. Only the air buoyancy effect caused by temperature is considered, while the airflow disturbance caused by infiltration and other human factors are ignored;
4. The wall thickness is far less than the height of the wall, and the heat conduction in a wall is considered one-dimensional heat conduction;
5. The indoor temperature changes caused by human body heat dissipation, furniture heat dissipation, and cold air infiltration are ignored.

### Model parameters setting

The numerical simulation was carried out using the ANSYS 16.0 software. The physical parameters of material and air are shown in Tables 1 and 2, respectively.

**Table 1:** Physical parameters of material.

	Material	Density (kg/m^3^)	Specific heat (J/kg·K)	Coefficient of thermal conductivity (W/m·K)
Porous wall	Porous ceramic	2418	3308	1.032
Wall	Concrete	2250	837	1.5
Glass cover	Glass	2500	840	0.76
Ground	Soil	2300	1178.2	2.36

**Table 2:** Physical parameters of air.

Temperature (K)	Density (kg/m^3^)	Specific heat (J/kg·K)	Coefficient of thermal conductivity (W/m·K)
273.15	1.293	1005	0.0244
283.15	1.247	1005	0.0251
293.15	2.205	1005	0.0258
303.15	1.165	1005	0.0267
313.15	1.128	1005	0.0275
323.15	1.093	1005	0.0282
333.15	1.060	1005	0.0289

During simulation, outdoor environmental parameters are measured during January in Nanyang, China. The DO model is used as a solar radiation model due to its semi-transparent characteristic^29,30^. The parameters of solar radiation are shown in Table 3.

**Table 3:** Parameters of solar radiation.

Solar incidence angle			Direct radiation intensity (W/m^2^)	Scattered radiation intensity (W/m^2^)		Scattered on the ground (W/m^2^)
X	Y	Z	Horizontal plane	Horizontal plane	Vertical plane	61.54
−0.81	0.08	0.58	952.70	67.61	60.79	

When there is solar radiation, vent 1 is opened, and the speed of fresh air is 0.3 m/s. When there is no solar radiation, vent 2 is opened, and the fresh air speed is 0.1 m/s. The fresh air temperature and free flow boundary temperature use outdoor air temperature as shown in Table 4.

**Table 4:** Hourly outdoor air temperature.

Time	1:00	2:00	3:00	4:00	5:00	6:00	7:00	8:00
Temperature (K)	273.85	273.25	272.85	272.55	272.35	272.15	271.75	271.55
Time	9:00	10:00	11:00	12:00	13:00	14:00	15:00	16:00
Temperature (K)	271.45	272.45	273.95	275.35	276.55	277.65	278.55	279.25
Time	17:00	18:00	19:00	20:00	21:00	22:00	23:00	24:00
Temperature (K)	279.35	278.85	277.55	276.75	275.85	275.05	274.75	274.25

The simulation procedure is summarized as follows: compiling the data in Table 4 into a profile-1 file, and then importing this file into the ANSYS-FLUENT software for calculation. The specific content of the profile-1 file is as follows:

((free-temperature-profile transient 24 0)

(time

0 3600 7200 10800 14400 18000 21600 25200 28800 32400 36000 39600 43200 46800 50400 54000 57600 61200 64800 72000 75600 79200 82800 86,400)

(temperature

273.85 273.25 272.85 272.55 272.35 272.15 271.75 271.55 271.45 272.45 273.95 275.35 276.55 277.65 278.55 279.25 279.35 278.85 277.55 276.75 275.85 275.05 274.75 274.25))

### Boundary conditions and initial conditions setting

To study the heating performance of the double-channel porous wall, only the south wall participates in the solar ray tracking model. The glass cover uses a mixed boundary condition. The boundary settings of the glass cover are presented in Table 5.

**Table 5:** Boundary settings of the glass cover.

Convective heat transfer coefficient (W/m^2^·K)	Free flow temperature (K)		External emissivity	External temperature (K)		Internal emissivity
13.68	276.55		0.94	276.55		0.85
Boundary types	Absorption rate			Transmittance		
	Visible light	Infrared	Hemispherical scattering coefficient	Visible light	Infrared	Hemispherical scattering coefficient
Translucent	0.04	0.04	0.04	0.88	0.88	0.88

The other walls of the room only consider convective heat transfer with the external environment. The surface heat transfer coefficient is 23.26 W/m^2^·K. The free flow temperature is the ambient temperature. The boundary parameters setting of the porous wall is shown in Table 6.

**Table 6:** Boundary parameters setting of porous wall.

	Infrared absorption	Visible absorptivity	Emissivity
Inner surface	–	–	0.87
Outer surface	0.9	0.95	0.87

Fluid–structure coupling boundary conditions are applied to the inner and outer surface of the heat storage wall and the inner surface of the vent. The ground uses fixed wall temperature boundary condition. The fresh air inlet uses velocity inlet boundary condition. The velocity is 0.3 m/s and the temperature is 276.55 K. The fan uses the fan boundary condition. The outlet uses the outflow boundary condition. The boundary conditions of the vent are shown in Table 7. The automatic saving is set to every 40 steps. The initial temperature is 276.55 K and the mode is global initialized.

**Table 7:** Boundary conditions of the vent.

	Daytime 08:00–18:00	Nighttime 18:00–08:00
Boundary types	Interior	Adiabatic wall
Vent state	Open	Close

The RNG k–ε turbulence model shows an improving accuracy and can be used for low Reynolds number fluid flow. Hence, the RNG k-ε model was used in the study^31,32^. The SIMPLEC algorithm^33^ and second-order upwind format were used to calculate the momentum, energy, radiation, and turbulence equations. In this study, the air is assumed to be incompressible fluid and the wind pressure effect is neglected. In addition, the PRESTO format and Green-Gauss Node Based format were used for pressure interpolation and gradient, respectively. The relaxation factors setting are presented in Table 8.

**Table 8:** The values of the relaxation factor.

Control items	Value
Pressure	0.3
Density	1.0
Body forces	0.5
Momentum	0.2
Turbulent kinetic energy	0.6
Turbulent dissipation rate	0.6
Turbulent viscosity	0.8
Energy	0.8
Discrete ordinates	0.8

The convergence conditions are summarized as follows:

1. The energy and radiation residuals should be less than 10^–6^ while other residuals should be less than 10^–3^;
2. The outlet temperature of the fan and indoor average temperature cannot change with the iteration for a long time. Thus, this is considered a convergence state;
3. The mass and energy balance error of the system should be less than 0.1%.

### Meshing and model validation

**Figure 2:**
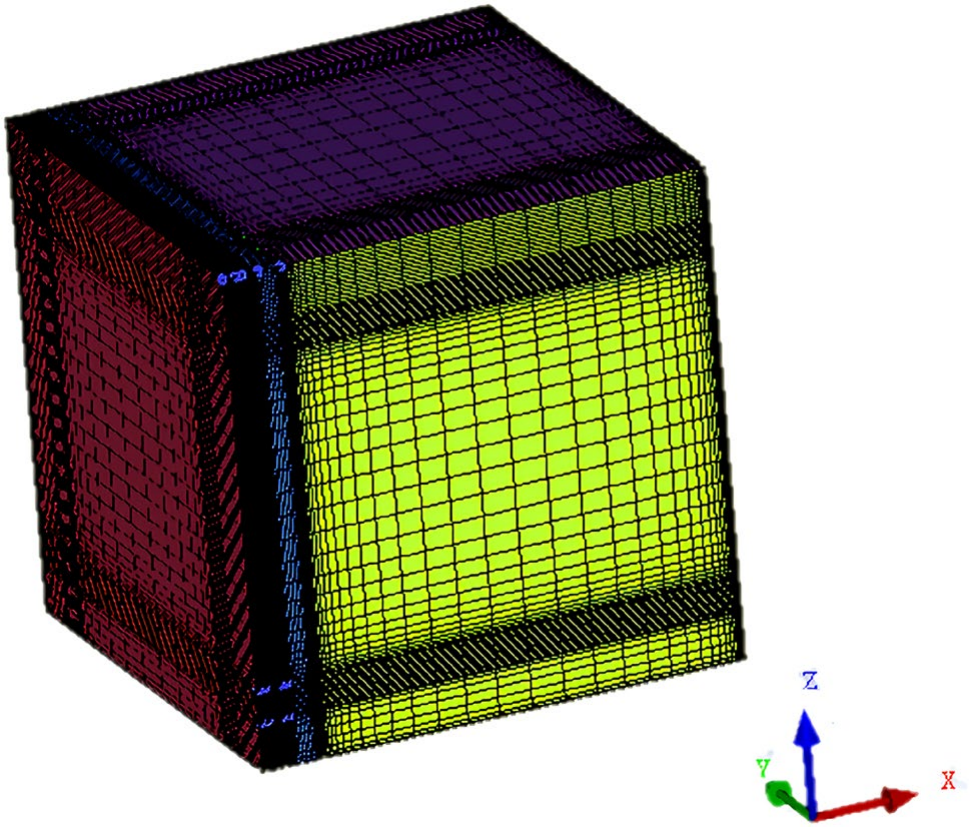
Mesh model.

The ANSYS ICEM was used for model grid division. To improve the mesh quality and convergence ability, the structured mesh was used. In this model, the positive directions of the X-axis, Y-axis, and Z-axis are respectively the north, the west, and the sky. Considering the complexity of the turbulent flow near the double-channel porous solar wall and the heating room, grid meshing was carried out by controlling the height of the grid on the first layer of the wall, in which the grid change rate was 1:1. To improve the calculation accuracy, the grid number of the vent and their vicinity were increased. The result of meshing is shown in Fig. 2, and the total number of grids is 530,000.

To evaluate the validity of the CFD model, the simulation results are compared to the results reported by Zhou et al^34^. The solar thermal efficiency (η) of the double-channel porous solar wall can be computed as:

8 $$\eta = \frac{Q}{IA}$$

where *A* is the effective absorbing area, *I* is the solar radiation intensity, and *Q* is the heat storage.

The relative error (RE) between the simulation values and experimental results is calculated as:

9 $$RE = \left| {\frac{{X_{{{\text{ref}}}} - X_{sim} }}{{X_{ref} }}} \right| \times 100\%$$

**Figure 3:**
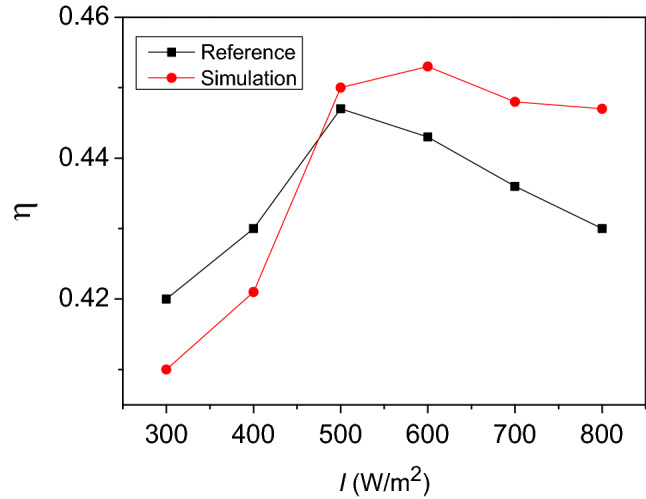
Comparison of thermal efficiency.

Figure 3 shows a comparison of thermal efficiency between the reference and simulation results. The results show that the maximum relative error is less than 5%. This means that CFD predicted data are consistent with the reference’s results. Hence, the proposed CFD model can be used in numerical simulation.

## Results and discussion

### Structure optimization of double-channel porous solar wall

The double-channel porous solar wall absorbs solar radiation and then stores heat energy in porous wall and thermal storage wall. Most of the heat energy is used for indoor heating, and the remaining heat energy is dissipated to the environment through glass cover. Therefore, the indoor temperature and heat storage are crucial factors when selecting the optimal structure for double-channel porous solar wall.

Heat storage is an important indicator to characterize the thermal performance of double-channel porous solar wall. The heat storage is calculated by internal and external wall surface temperature. According to assumed conditions, the thermal conductivity of the wall can be regarded as one-dimensional steady thermal conductivity along with the thickness of the wall, and the heat storage can be computed as:

10 $$Q = \rho cV(t_{1} - t_{2} )$$

where *ρ* is the density of the wall [kg/m^3^], *c* is the specific heat capacity of the wall [J/kg·K], *V* is the volume of the wall [m^3^], *t*_1_ is the wall temperature of outer surface [K], and *t*_2_ is the wall temperature of inner surface [K].

The thickness of the porous wall plays an important role in the heating performance of the room. Therefore, the impacts of different values of the porous wall (δ_2_), the air channel 1 spacing (δ_1_), and the air channel 2 spacing (δ_3_) on the indoor temperature and heat storage are studied. The prerequisites of this study are:

1. Keep the sum of δ_1_, δ_2_, and δ_3_ at a constant value of 0.22 m;
2. The values of δ_1_ and δ_3_ are between 0.04 m and 0.10 m;
3. The values of δ_2_ are between 0.04 m and 0.12 m.

Under control conditions, the calculation results are summarized in Table 9.

**Table 9:** Calculation results of different structures.

Group	δ_1_ (m)	δ_2_ (m)	δ_3_ (m)	T_in_ (K)	Q_p_ (MJ)	Q_h_ (MJ)	Q (MJ)
1	0.04	0.12	0.06	288.05	156.36	11.65	168.01
2	0.06	0.12	0.04	288.40	154.55	12.36	166.91
3	0.04	0.10	0.08	288.62	137.28	11.72	149.00
4	0.06	0.10	0.60	288.68	136.42	11.86	148.28
5	0.08	0.10	0.40	288.75	133.83	12.32	146.15
6	0.04	0.08	0.10	288.86	95.31	12.39	107.70
7	0.06	0.08	0.08	288.93	92.61	13.26	105.87
8	0.08	0.08	0.06	289.86	86.50	13.67	100.17
9	0.10	0.08	0.04	289.90	85.87	13.91	99.78
10	0.06	0.06	0.10	289.82	64.62	13.19	77.81
11	0.08	0.06	0.08	289.93	60.47	13.72	74.19
12	0.10	0.06	0.06	290.05	60.34	14.00	74.34
13	0.08	0.04	0.10	290.60	28.77	14.26	43.03
14	0.10	0.04	0.08	290.77	28.33	14.50	42.83

Note: T_in_ Indoor temperature, Q_p_ Heat-storage of porous wall, Q_h_ Heat-storage of thermal storage wall, Q Total heat storage.

It can be seen that, when δ2 is 0.08 m, the heat storage of porous wall decreases from 95.31 MJ to 85.87 MJ while δ1 increases from 0.04 m to 0.1 m. At the same time, the heat storage of thermal storage wall increases from 12.39 MJ to 13.91 MJ. The main reason for this variation is the fact that the fresh air takes away more heat from the porous wall. According to the indoor air quality standard of China, the demand range of indoor temperature ranges between 289.15 K and 303.15 K in winter^35^. Moreover, the higher the heat storage of the wall, the better it is for indoor heating. The temperature value of group 1 to group 7 is below 289.15 K, which does not meet the requirement of indoor temperature. The total heat storage of group 8 is higher than those of group 9 to group 14. Therefore, it is believed that group 8 is the optimal structure of double-channel porous solar wall under control conditions.

### Influence of the external surface emissivity on the double-channel porous solar wall

**Figure 4:**
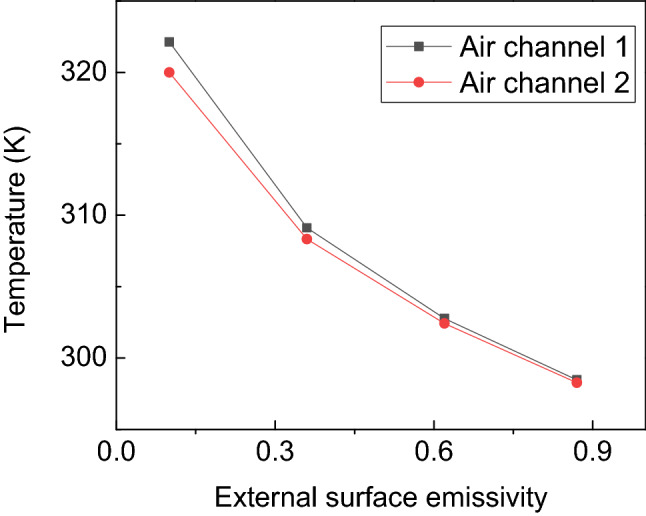
The influence of the external surface emissivity on the temperature of air channel.

The influence of the external surface emissivity of porous wall on the temperature of air channel 1 and air channel 2 is shown in Fig. 4. The temperature of air channel 1 decreases from 322.14 K to 298.46 K while the emissivity increases from 0.1 to 0.87. Similarly, the temperature of air channel 2 decreases from 320 K to 298.27 K. This is due to the heat losses from the outer surface of the porous wall that increases with the increase of the emissivity. This leads to the decrease of the convective heat transfer between porous wall and air channel 1. From the above analysis, it can be concluded that the temperature of the air channel decreases with the increase of emissivity.

**Figure 5:**
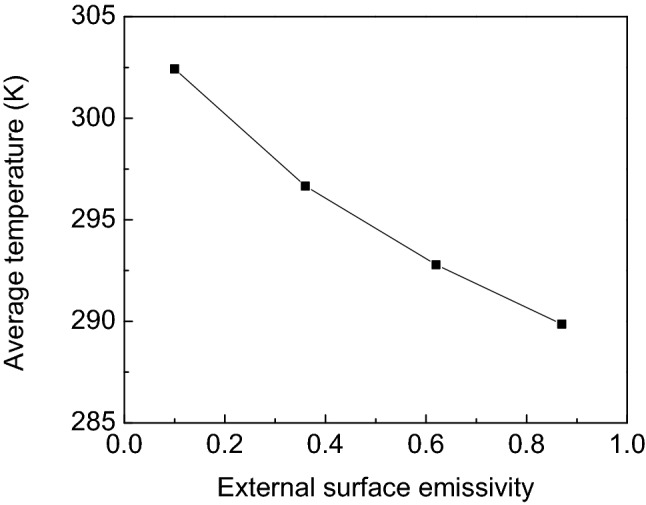
Effect of the external surface emissivity of the porous wall on indoor average temperature.

The effect of the external surface emissivity of the porous wall on indoor average temperature is shown in Fig. 5. It can be seen that the average indoor temperature of the room decreases from 302.43 K to 289.86 K when the external surface emissivity of the porous wall increases from 0.1 to 0.87. This is due to the fact that the air sent to the indoor room obtained less heat energy from the porous wall as well as the thermal storage wall, which eventually causes the indoor average temperature to decrease. Thus, it can be concluded that the external surface emissivity of the porous wall has a significant effect on indoor average temperature.

**Figure 6:**
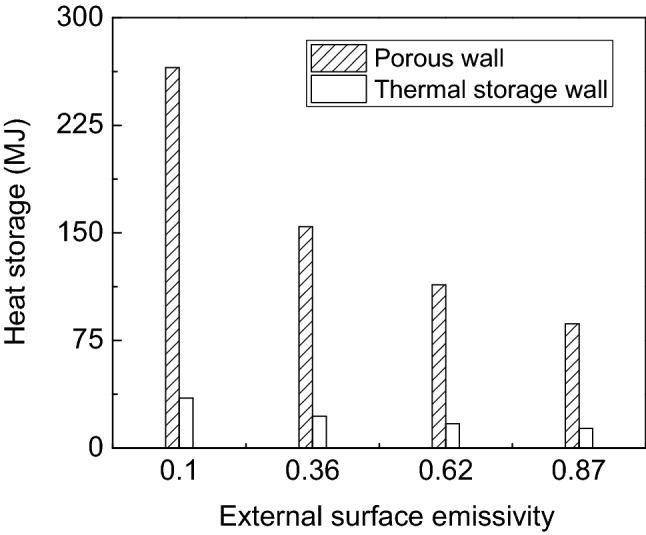
The influence of the external surface emissivity on the heat storage of porous wall and thermal storage wall.

Figure 6 shows the influence of the external surface emissivity on the heat storage of porous wall and thermal storage wall. It is clear that the heat storage of porous wall decreases from 265.26 MJ to 86.5 MJ while the emissivity increases from 0.1 to 0.87, which indicates that the higher the emissivity of the porous wall, the more radiant energy is released to the environment through the glass cover. This leads to less heat being stored in porous wall. On the contrary, the heat stored in the thermal wall slightly decreases with the increase of emissivity. From the above analysis, it can be concluded that the emissivity of the porous wall has a significant impact on the heat storage of the porous wall and little effect on the thermal storage wall.

### Analysis of temperature field of porous wall

**Figure 7:**
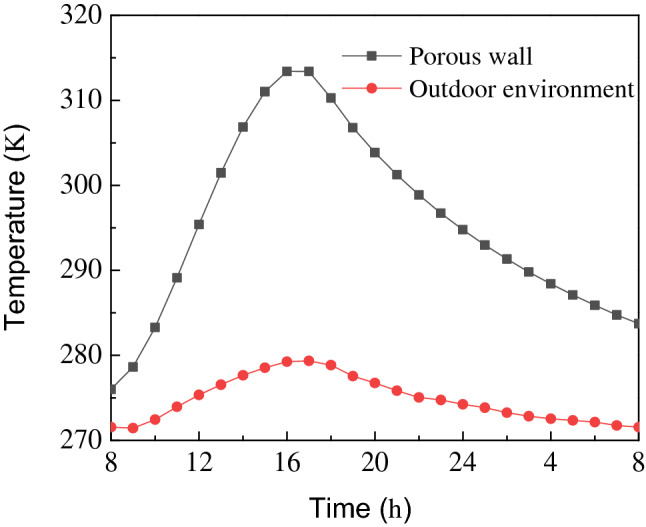
The temperature change of porous wall with time.

The temperature change of porous wall function over time is shown in Fig. 7. Initially, the temperature of the porous wall shows a sharp increase with time and then reaches the highest temperature of 313.40 K at 16:00. Afterwards, the temperature starts to decrease till the next day at 8:00. In this process, the maximum temperature difference between the porous wall and the outdoor environment is 34.15 K, which indicates that the porous wall absorbs a large quantity of solar radiation energy before 16:00. When the sun’s radiation starts to fade, the porous wall absorbs less solar radiation. At night, air channel 1 is closed and the heat stored in the porous wall is transmitted to the room by convective heat transfer. The temperature of the porous wall drops to 283.72 K on the following day, and the minimum temperature difference between the porous wall and the outdoor environment is 12.17 K. The temperature of the porous wall is still higher than that of the outdoor environment at night, which indicates that the heat stored in the porous wall can be used for heating. Therefore, it can be concluded that porous wall plays a crucial role in heat preservation at night.

**Figure 8:**
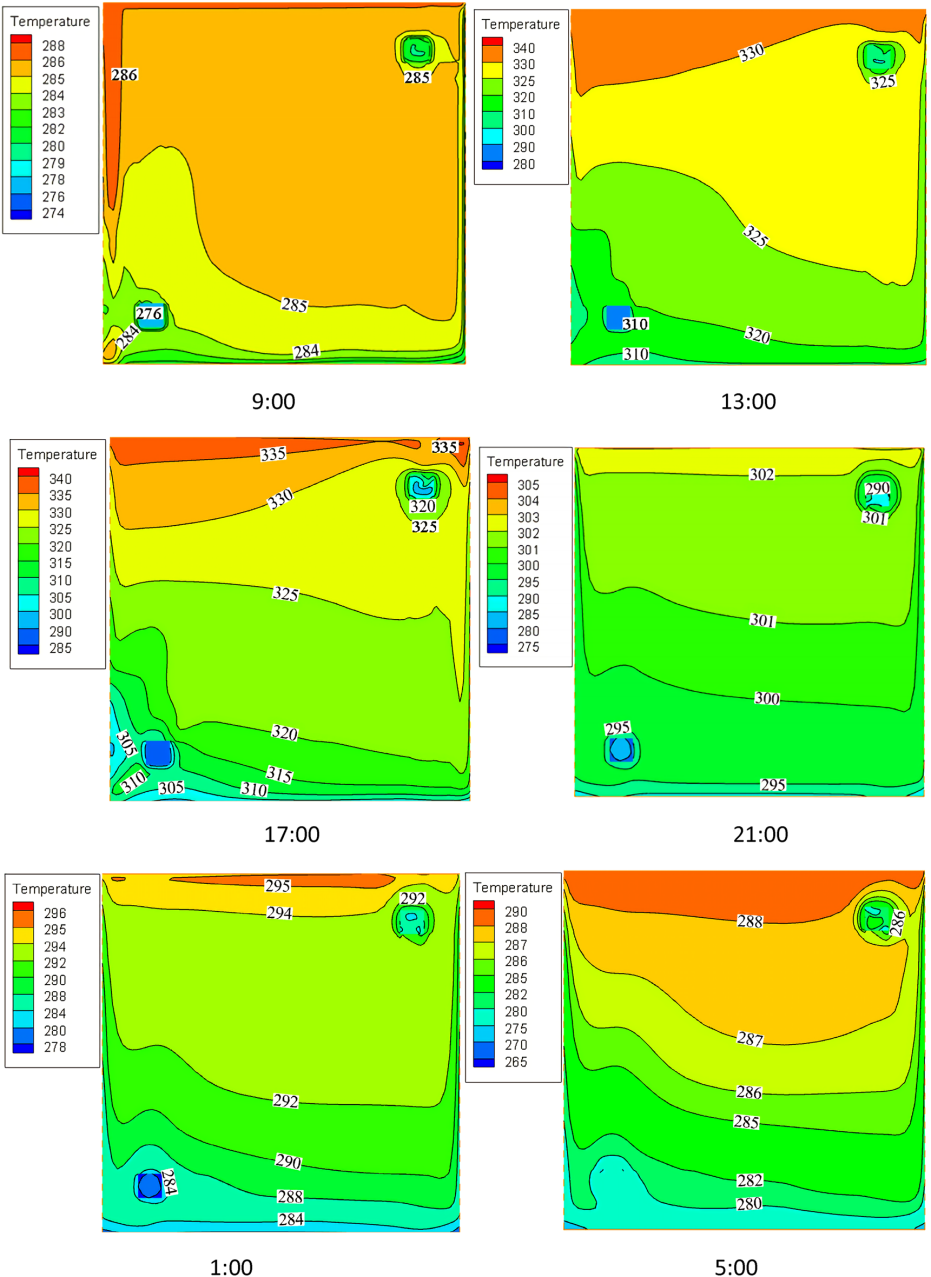
The temperature field of the outer surface of the porous wall at different times.

To investigate the internal temperature change of the porous wall, the temperature distribution of the outer surface was observed every 4 h. The temperature field of the outer surface of the porous wall at different times is illustrated in Fig. 8. The bottom left of the figure shows the temperature distribution near vent 3 and the top right of this figure shows the temperature distribution near vent 4. It is clear that the temperature of the upper part is higher than that of the lower part, independently of the time factor. A more reasonable explanation for this phenomenon is that air becomes less dense as it continues to absorb heat from the porous wall when it moves upward under the action of buoyancy and fan. This demonstrates that the bulk of heat entering the room comes from the porous wall.

### Analysis of temperature field of thermal storage wall

**Figure 9:**
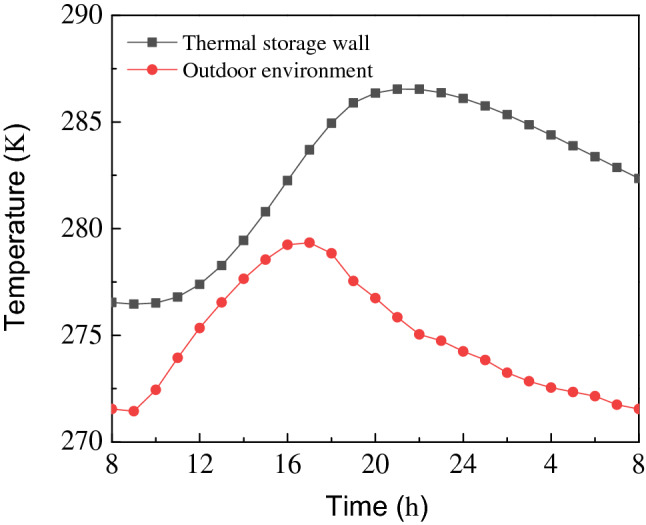
The average temperature change of the thermal storage wall with time.

The thermal storage wall is located between the porous wall and the room. It is used for storing redundant heat in the daytime and releasing heat into a room at night. The average temperature change of the thermal storage wall with time is illustrated in Fig. 9. It can be seen that the average temperature of the thermal storage wall increases from 276.55 K to 286.55 K and then starts to decrease. In addition, the outdoor environment temperature has a similar variation trend concerning the average temperature of thermal storage walls.

**Figure 10:**
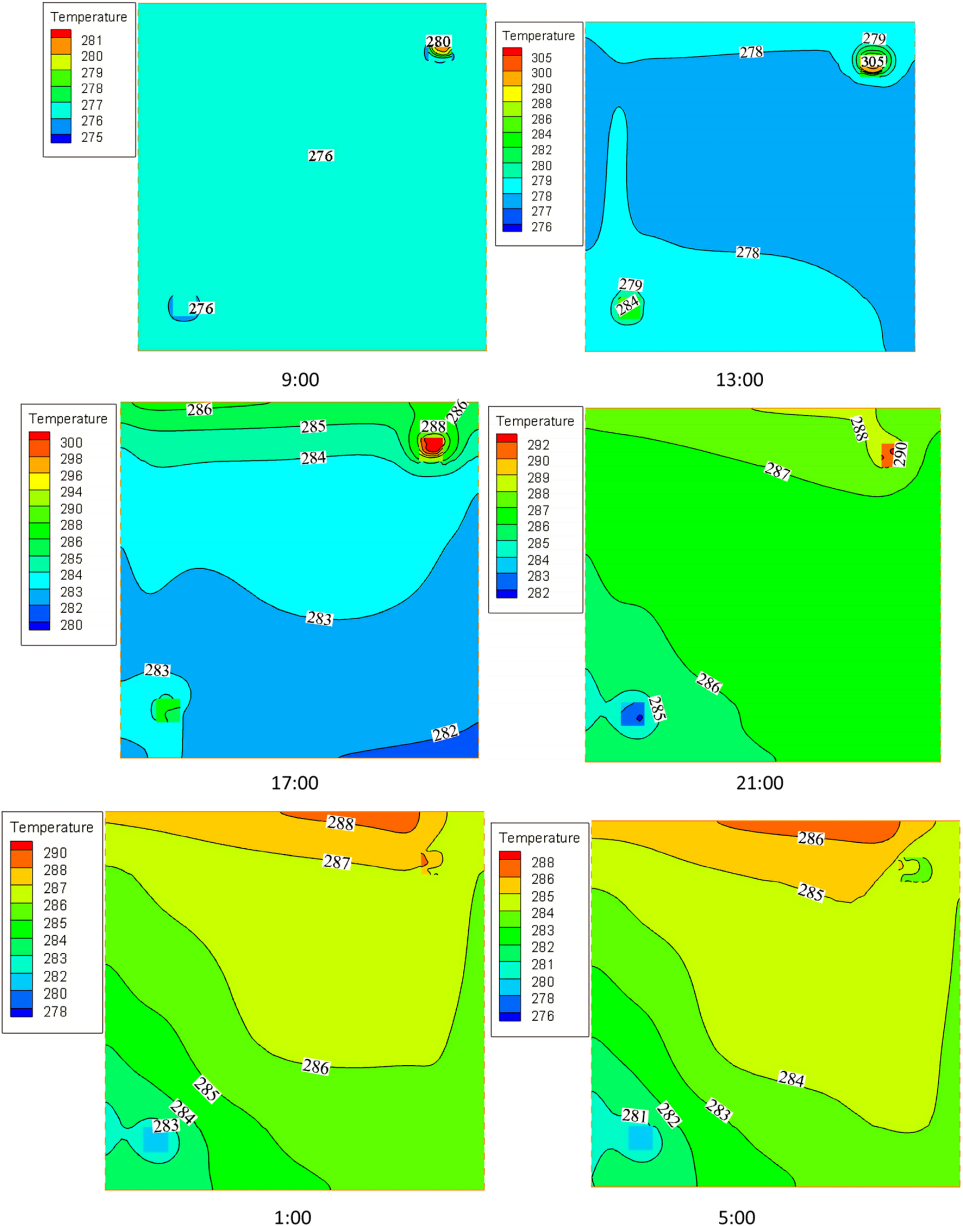
The internal temperature field of the thermal storage wall at different times.

The internal temperature field of the thermal storage wall at different times is shown in Fig. 10. Accordingly, the temperature of the thermal storage wall shows an upward trend from 9:00 to 21:00 and then starts to decrease. This is due to the fact that in the daytime, the porous wall transfers excess heat energy to the thermal storage wall in the form of radiation and convection heat transfers. This causes the temperature of the thermal storage wall to increase. After air channel 1 is closed at night, circulating air is sent to the room by air channel 2. From 21:00 to 5:00, the heat energy in the thermal storage wall continues to be carried away by circulating air for heating, which leads to the slow decrease of the temperature of the thermal storage wall.

## Conclusions

The present work proposes a new kind of double-channel porous solar wall. This type of wall not only enhances the heat exchange between the wall and the air, but also introduces more fresh air from the outside to keep the indoor air fresh. In this study, a different 3D model of a double-channel solar wall was built up to obtain the optimal structure. The wall structure optimization and thermal performance of double-channel porous solar walls are carried out by CFD method. Based on the optimal structure, the influence of the external surface emissivity on the heating characteristics of a double-channel porous solar wall is discussed. At the same time, the temperature field of the porous wall and thermal storage wall is analyzed. The following conclusions can finally be drawn.

1. Under the current conditions of this study, the optimal structure parameters are summarized as follows: the air channel 1 spacing is 0.08 m, the porous wall thickness is 0.08 m, and the air channel 2 spacing is 0.06 m.
2. The temperature of air channel 1 and air channel 2, the indoor temperature, and the heat storage of the porous wall decrease with the increase of the surface emissivity of the porous wall. The emissivity of the porous wall has a significant impact on the heat storage of the porous wall and little effect on the thermal storage wall.
3. In 24 h, the porous wall and thermal storage wall show a trend of first increasing and then decreasing, while the temperature of the porous wall is always higher than that of the outdoor environment. This means that the amount of heat stored in a porous wall can provide plenty of heat to keep the room warm at night.

The wall structure has a great influence on the heat storage of the wall, and the optimal wall structure can be obtained through the optimization calculation. Reasonable wall structure can store enough heat storage and reduce the construction cost, which is conducive to its application in practical projects. In addition, the emissivity of the wall surface can also affect the heat storage of the wall at night, and the external surface of the porous wall releases thermal energy to the environment, resulting in energy loss. To improve the heat storage performance of a double-channel porous solar wall, the outer surface of the porous wall should use a lower emissivity material. Due to the lack of experiment conditions, an experimental study on the thermal performance of double-channel porous solar walls has not been conducted. A comprehensive experiment and analysis of energy loss, indoor temperature, the cost of material, and applicability of double-channel porous solar wall will be carried out in the subsequent study.

## Data Availability

All datasets on which the conclusions of the manuscript rely are mentioned or presented in the main paper.
